# Anatomic structure of the internal iliac artery and its educative dissection for peripartum and pelvic hemorrhage

**DOI:** 10.4274/tjod.23245

**Published:** 2018-06-21

**Authors:** İlker Selçuk, Murat Yassa, İlkan Tatar, Emre Huri

**Affiliations:** 1 University of Health Sciences, Zekai Tahir Burak Woman’s Health Health Practice and Research Center, Clinic of Gynecologic Oncology, Ankara, Turkey; 2 İstanbul Fatih Sultan Mehmet Training and Research Hospital, Clinic of Obstetrics and Gynecology, İstanbul, Turkey; 3 Hacettepe University Faculty of Medicine, Department of Anatomy, Ankara, Turkey; 4 Hacettepe University Faculty of Medicine, Department of Urology, Ankara, Turkey

**Keywords:** Internal iliac artery, dissection, postpartum, hemorrhage, obstetrics

## Abstract

The abdominal aorta is divided into two parts (right and left) at the level of the fourth-fifth lumbar vertebra and called the common iliac artery. Anterior to the sacroiliac joint, common iliac arteries are divided into external and internal iliac arteries. The external iliac artery supplies the lower limb, and the internal iliac artery is the major vascular supply of the pelvis. Internal iliac artery is divided into anterior and posterior trunk. The anterior trunk supplies the pelvis, visceral organs, and the posterior trunk supplies pelvic parietal structures. The broad ligament envelopes the uterus anteriorly and posteriorly with its sheets and continues as the pelvic peritoneum at the lateral side of the pelvic wall. After cutting the pelvic peritoneum, the retroperitoneal area is visualized and the internal iliac artery with other great vessels of the abdomen can be noted.

## Introduction

**Figure 1:**
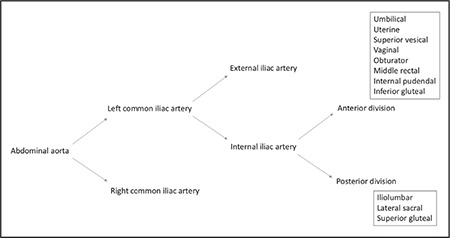
Branches of internal iliac artery

**Figure 2:**
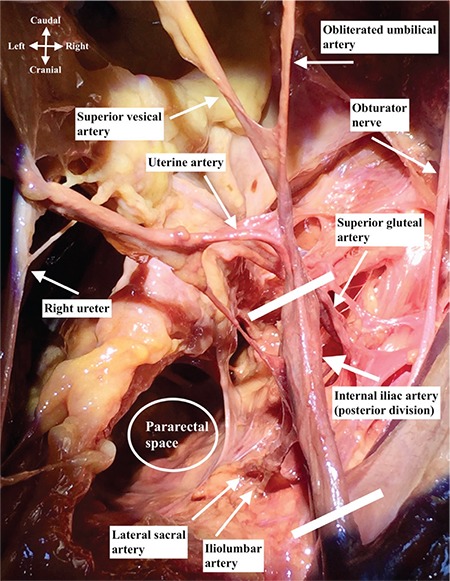
Right internal iliac artery dissection, superior view

The abdominal aorta is divided into two parts (right and left) at the level of the fourth-fifth lumbar vertebra and called the common iliac artery. Anterior to the sacroiliac joint, the common iliac arteries are divided into external and internal iliac arteries. The external iliac artery supplies the lower limb and internal iliac artery is the major vascular supply of the pelvis^(1)^. The internal iliac artery is one of the two divided parts of the common iliac artery, it passes medially over the pelvic brim and runs downward to the pelvic cavity. At the upper margin of the greater sciatic foramen, it is divided anteriorly and posteriorly. The ureter stands at the medial aspect of internal iliac artery, the pararectal space is noted between the ureter and the internal iliac artery. It is the main blood supply to the pelvic organs, gluteal muscles, and perineum, with the anterior (visceral supply) and posterior (parietal supply) trunks. There is an important and increased potential of anatomic variations for the internal iliac artery, especially for the end branches, so the pelvic surgeon should be careful during the dissection of this area. Figure 1 and Figure 2 demonstrates the branches of the internal iliac artery.

**Figure 3:**
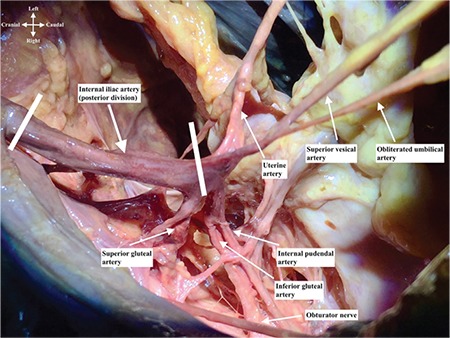
Right internal iliac artery dissection, lateral view

**Figure 4:**
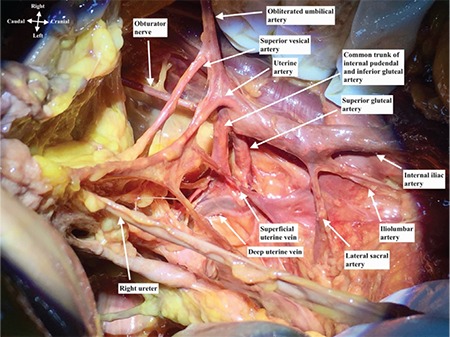
Right internal iliac artery dissection, medial view

Anterior trunk of internal iliac artery runs anteriorly along the lateral pelvic wall and supplies most of the pelvic viscera. The arteries are the umbilical (obliterated), uterine, superior vesical, vaginal, obturator, middle rectal, internal pudendal, and inferior gluteal arteries^(2)^. The posterior trunk of the internal iliac artery runs posteriorly to the pelvic wall and gluteal region^(3)^. The arteries are as follows: the iliol umbar, which anastomoses with the superior gluteal and circumflex iliac arteries; the lateral sacral, which anastomoses with the median sacral artery; and the superior gluteal artery (Figure 3, lateral view of right internal iliac artery after dissection), which anastomoses with the lateral sacral, inferior gluteal and internal pudendal arteries (Figure 4). The superior gluteal artery is the main part and continuation of posterior trunk and sometimes it may arise directly from the internal iliac artery. It runs between the lumbosacral trunk and first sacral nerve.

## Materials and Methods

This cadaveric dissection was performed at the “Pelvic Reconstructive and Functional Urology Surgery Cadaveric Workshop”; an advanced masterclass course on anatomy and surgery by using fresh frozen cadavers on 22-23 April 2017 at Bahçeşehir University Faculty of Medicine, Prof. Rhoton Anatomy Laboratory, İstanbul/Turkey.

## Arteries of anterior division

### Obliterated umbilical artery

It is the blind end of the internal iliac artery (Figure 2, superior view of right internal iliac artery after dissection) and an important anatomic landmark to identify the uterine artery, especially during laparoscopic procedures. Moreover, it is in close relation with paravesical space and it divides the paravesical space into two parts. When traction is applied where it attaches to the anterior abdominal wall, it may indicate the uterine artery. The superior vesical artery arises from the proximal part of the umbilical artery, which supplies the upper portion of the bladder. The inferior vesical artery supplies the base of the bladder; however, it is not possible to detect it during every dissection every dissection. It is also called the vaginal artery. The umbilical artery becomes the medial umbilical ligament when it reaches the anterior abdominal wall.

### Uterine artery

The uterine artery arises from the anterior division of the internal iliac artery and goes antero-medially to the lateral part of uterine cervix below the isthmic part of uterus, where it crosses the ureter superiorly (Figure 4, medial view of right internal iliac artery after dissection). It begins between the origins of the obturator and umbilical arteries; however, sometimes it may have a common trunk with the superior vesical artery. The uterine artery runs between the anterior and posterior layer of broad ligament, above the cardinal ligament. It anastomoses with the ovarian artery and also gives a vaginal branch.

### Clinical tip: how to identify the uterine artery?

Downward dissection from the obliterated umbilical artery is one way to identify the uterine artery. Another method is to follow the ureter to the point where it crosses under the uterine artery. Moreover, after opening the retroperitoneal region on the pelvic sidewall, antero-medial dissection of internal iliac artery may show the uterine artery.

### Obturator artery

The obturator artery runs antero-infero-laterally on the obturator fascia and exits from the pelvis through the obturator foramen. The origin of the obturator artery varies; however, it mostly arises near the origin of the umbilical artery. It goes just under the obturator nerve and anastomoses with the inferior epigastric artery or external iliac artery via the pubic branch (corona mortis); in the pelvis, the obturator artery gives rise to the pubic branch it leaves the pelvis from the obturator foramen^(4)^.

### Clinical tip: obturator artery

An aberrant (accessory obturator artery), a variation, may arise from the external iliac artery and runs to the obturator foramen.

### Clinical tip: corona mortis

Posterior to the superior pubic ramus at the superior part of the paravesical space, a vascular connection between the external iliac artery or inferior epigastric artery and the obturator artery can be detected. These vascular connections are mostly venous; however, the surgeon should be careful, especially for arterial anastomoses^(5)^.

### Internal pudendal artery

**Figure 5:**
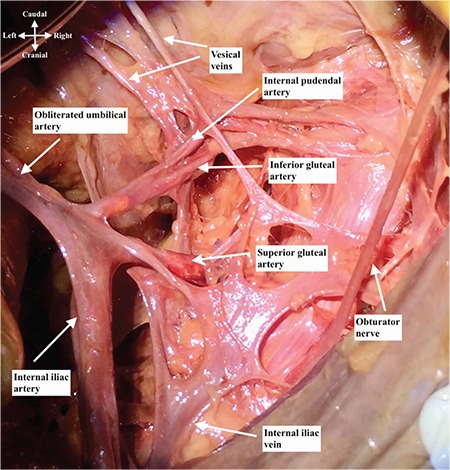
Right internal iliac artery dissection over the internal iliac vein, lateral view

This artery runs infero-laterally, anterior to the piriformis muscle and sacral plexus (Figure 5). It passes through the greater sciatic foramen along the posterior aspect of the ischial spine close to the sacrospinous ligament and enters the ischio-anal fossa through the lesser sciatic foramen. Injury of the internal pudendal artery could be managed by compression on the ischiorectal/ischioanal fossa. The internal pudendal artery gives rise to the dorsal artery of clitoris.

### Inferior gluteal artery

This passes between the sacral nerves, usually S1 and S2, sometimes S2 and S3 and leaves the pelvis through the greater sciatic foramen below the piriformis muscle (Figure 5). The inferior gluteal artery is also a danger zone during sacrospinous ligament fixation^(6)^.

### Superior vesical artery

It goes antero-inferiorly from the anterior division of internal iliac artery (sometimes it may arise from the same origin with uterine artery or from the obliterated umbilical artery) and supplies the distal end of the ureter and bladder.

### How to dissect the internal iliac artery?

- The broad ligament envelopes the uterus anteriorly and posteriorly with its sheets and continues as the pelvic peritoneum at the lateral side of the pelvic wall.

- After cutting the pelvic peritoneum, the retroperitoneal area is visualized, the incision is extended to the paracolic area parallel to the infundibulopelvic ligament.

- The posterior sheet of the broad ligament is pulled medially, where the ureter and infundibulopelvic ligament lie.

- The common iliac artery is identified over the sacroiliac joint (the ureter crosses over it).

- When the adipose and lymphatic tissue is retracted softly over the vessels, the internal iliac artery is identified over the pelvic brim. 

- The internal iliac artery first goes downwards, then straight to the anterior abdominal wall where it ends as the umbilical artery. 

- When the adipose and lymphatic tissue is dissected over the internal iliac artery, the posterior division is identified at the first 3-4 cm, after this part, the anterior division starts and ligation of internal iliac artery could be performed at this point.

- For intractable pelvic hemorrhage the ligation of the internal iliac artery should be performed at the beginning of anterior division.

- Care should be taken with the external iliac vein, where it lies at the lateral part of the artery.
